# Impact of the secondary plant metabolite Cucurbitacin B on the demographical traits of the melon aphid, *Aphis gossypii*

**DOI:** 10.1038/s41598-018-34821-w

**Published:** 2018-11-07

**Authors:** Hafiz Kamran Yousaf, Tisheng Shan, Xuewei Chen, Kangsheng Ma, Xueyan Shi, Nicolas Desneux, Antonio Biondi, Xiwu Gao

**Affiliations:** 1 Department of Entomology, College of Plant Protection, China Agricultural University, Beijing, 100193 China; 2 INRA (French National Institute for Agricultural Research), Université Côte d’Azur, CNRS, UMR 1355-7254, Institute Sophia Agrobiotech Sophia Antipolis, France; 3 Department of Agriculture, Food and Environment, University of Catania, Catania, Italy

## Abstract

Cucurbitacin B is a natural triterpene present in plants of Cucurbitaceae family, which are among the host plants for melon aphid, *Aphis gossypii*. In present study we characterized the effects of two cucurbitacin B concentrations on the biological parameters of adults (F_0_) and of juveniles and adults of their progeny (F_1_). The results showed that cucurbitacin B at 25 ppm significantly reduced the adult longevity and fecundity of both F_0_ and F_1_ generation. Exposure of F_0_ generation to 25 ppm though reduced the demographic traits of F_1_ including the intrinsic rate of increase *r* (day^−1^), generation time *T* (day), finite rate of increase *λ* (day^−1^), however, only net reproductive rate *R*_0_ (offspring/individual) decreased significantly. While 100 ppm reduced not only the longevity and fecundity of F_0_ generation but also the longevity of F_1_ generation. Fecundity of F_1_ was not affected by 100 ppm of cucurbitacin B, however, *R*_0_ (offspring/individual) and *T* (day) of F_1_ generation were lower than the control population. These results support the hypothesis that high contents of cucurbitacin B caused negative impact on melon aphid and could be used as a lead for classical selection of resistant varieties of plants that are main hosts for the melon aphid.

## Introduction

Plant secondary metabolites can serve as defensive compounds against herbivores (allelochemicals), though they are usually considered insignificant for regular plant growth processes, where primary metabolites play a crucial role^1^. Some plant materials categorizing as phenols, alkaloids, terpenes, flavonoids and other associated compounds, have repellent and/or antifeedant effects for phytophagous insects^2^. For example, alkaloids are feeding deterrents against *Leptinotarsa decemlineata* and *Empoasca fabae*^3^. Phenolic compounds in wheat act as deterrent for the cereal aphid, *Rhopalosiphum padi*^4^. High concentration of phenols in willow plant deterred the feeding of leaf beetle, *Galerucella lineola*^5^. Phenols together with flavonols are related to resistance to whitefly in black gram^6^. High level of gossypol in cotton plant prolonged the immature duration and reduced the survival and fecundity of *Aphis gossypii*^7^. Triterpenes, as major plant terpenoids, have remarkable properties, such as antibacterial^8^, analgesic^9^, neuroprotective^10^, antioxidant^11^, etc. In addition, some of the triterpenes, such as azadirachtin, exhibited clear repellent, antifeedant, growth and reproduction inhibiting effects, and even acute toxicity towards many insect species^12–15^.

Plant species belonging to the cucurbitaceae family contain several naturally related triterpenes, collectively known as cucurbitacins, such as cucurbitacin A, B, C, D, E, I, J, K and L^16,17^. Cucurbitacins are natural tetracyclic triterpene compounds in the plant of Cucurbitaceae family^18^. These are constitutive or insect-induced allelochemicals and have been shown to have exert acute and sublethal toxicity, as well as deterrents effects for feeding and oviposition in insects^19,20^. Ethanol extracts from the fruit of *Citrullus colocynthis* contained cucurbitacin E glycoside and caused mortality of the cowpea aphid, *Aphis craccivora*^21^. High level of cucurbitacin C in cucumber (*Cucumis sativus*) decreased the survival rate and population growth of spider mite (*Tetranychus urticae*) in cucumber plants^19,22^. Cucurbitacin D inhibited the reproduction of spider mites when applied on cotton cotyledon^23^. Cucurbitacin B not only deterred the european corn borer (*Ostrinia nubilalis*) and beet armyworm (*Spodoptera exigua*) from oviposition, but deterred feeding of yellow mealworm (*Tenebrio molitor*) and cinereous cockroaches (*Nauphoeta cinerea*)^20^. Cucurbitacin E and I could act as feeding inhibitor of flea beetle (*Phyllotreta nemorum*)^24^.

Melon aphid is a deleterious pest of numerous vegetable crops worldwide, especially Cucurbitaceae^25,26^. This pest causes direct and indirect damage to its host plants by removing the photoassimilates and transmitting the pathogenic viruses^27,28^, including those causing diseases for cucurbitaceae crop plants, e.g. cucumber mosaic virus, potyvirus and zucchini yellow virus to a wide range of crops and causes heavy loss of yields^29^. To control this pest in such crops, several insecticides have been developed and adopted into Integrated Pest Management (IPM) packages worldwide^30–33^. However, the use of insecticides has some drawbacks, which are known for their potential side effects on non-target organisms^34^, such as aphid natural enemies^35,36^, and for the development of insecticide resistance in aphids^37,38^. Therefore, non-chemical control strategies, such as biological control^39–41^, biopesticides^42,43^, and plant resistance should be prioritized for the sustainable control of aphid pests.

For an environmentally sound IPM programs, it is very important to understand the ecology of the insect pest. Insect life table provide a wide-ranging narrative of survival, development, life expectancy and fecundity of a population. Moreover, the age-stage life table is a reliable tool for studying sublethal effects at population levels^44^, and all these information are basic for understanding the ecology of the insect pests. However, the conventional age-specific life table was usually used for dealing with only the female age-specific population and neglected the different developmental rate and male population, which may cause inaccuracy in scheming the different demographic parameters i.e. net reproductive rate, intrinsic rate of increase and mean generation time. Chi & Liu (1985)^45^ and Chi (1988)^46^ developed an age-stage, two-sex life table to take the stage differentiation and the male population into consideration. Although there were only female *A*. *gossypii* in this study, to correctly analyze the population parameters, provide a correct description of stage differentiation, and to avoid problems inherent in female age-specific life tables^44^, we used the age-stage two-sex life table in our work.

It is known that high concentration of globe cucumber extract can cause high mortality and decrease the longevity of melon aphid adults^47^. However, the effects of the principal bioactive compounds of Cucurbitaceae plants have never been properly characterized for this important insect pest. In this context, we aimed at assessing the potential effects of cucurbitacin B, a principle secondary metabolite in plants of the Cucurbitaceae family^18,48^, on the main life history traits of *A*. *gossypii* at intra and transgenerational levels. This new knowledge can be crucial for understanding the potential plant-aphid interactions mediated by this important allelochemical, and also can supply the basic data for further application of cucurbitacin B for the integrated pest management of this pest.

## Results

### Acute toxicity of cucurbitacin B on melon aphid

**Figure 1:**
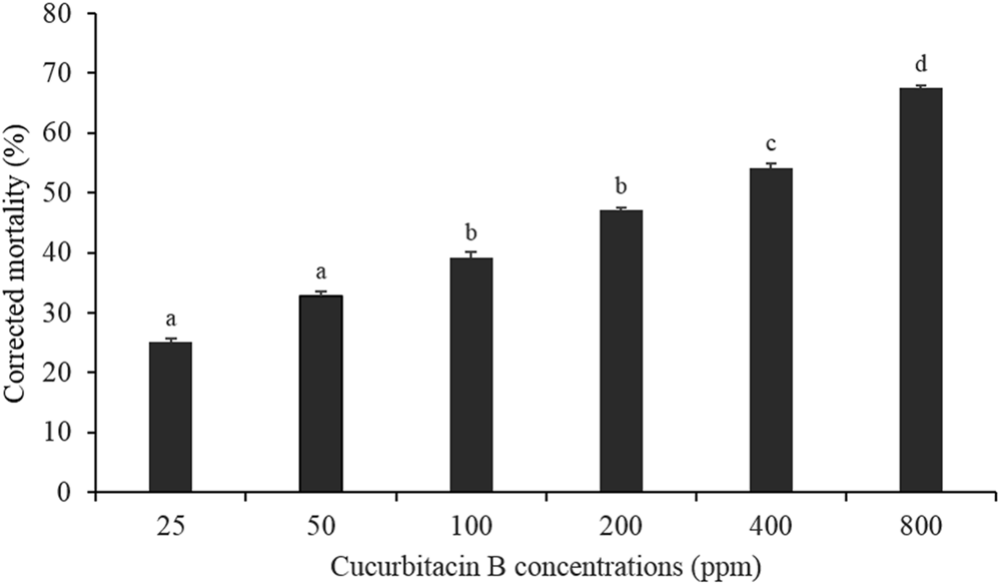
Corrected mortality of *A*. *gossypii* when adults were exposed to different concentrations of cucurbitacin B. Columns bearing different letters are significantly different at *P* < 0.05 (one-way ANOVA followed by Tukey HSD tests).

The mortality of *A*. *gossypii* feeding on artificial diet contaminated with the various concentrations of cucurbitacin B are shown in (Fig. 1). The mortality increased proportionally with the cucurbitacin B concentration increase. Cucurbitacin B at 800 ppm resulted in an aphid mortality of 67.47% mortality, while in the untreated control the mortality was 8.8% (*F* = 18.51; df 5,17; *P* < 0.001).

### Effects of cucurbitacin B on longevity and fecundity of parental (F_0_) and F_1_ generation of *Aphis gossypii*

Exposure of melon aphid to two concentrations of cucurbitacin B significantly suppressed the longevity (*F* = 24.68; df = 2, 10; *P* < 0.001) and fecundity (*F* = 33.06; df = 2, 10; *P* < 0.001) of the exposed population of F_0_ generation. Moreover, 100 ppm cucurbitacin B, exhibited a stronger effect than 25 ppm. The longevity of the F_1_ generation was significantly affected by both cucurbitacin B concentrations as compared to control group (*F* = 10.37; df = 2, 10; *P* < 0.001). However, the fecundity of the F_1_ generation was significantly decreased by 25 ppm of cucurbitacin B as compared to control (*F* = 5.18; df = 2, 10; *P* = 0.007), while it didn’t be affected by 100 ppm of cucurbitacin B (Table 1).

**Table 1:** Mean (±SE) values of developmental times of various life stages of aphids belonging to the F_1_ generation descending from parents (F_0_) and longevity and fecundity of F_0_ generation exposed to 25 and 100 ppm of cucurbitacin B, compared to the untreated control population.

Treatment	1^st^ instar	2^nd^ instar	3^rd^ instar	4^th^ instar	Total immature instars	Adult longevity F1	Adult Fecundity F_1_	Adult Longevity F_0_	Adult Longevity F_0_
Control	2.26 ± 0.08a	1.53 ± 0.07b	1.0 ± 0.03b	1.73 ± 0.07a	6.61 ± 1.09a	19.36 ± 0.65a	40.05 ± 2.41a	14.55 ± 0.52a	17.51 ± 1.13a
25 ppm	1.90 ± 0.07b	1.86 ± 0.08a	1.10 ± 0.03b	1.81 ± 0.09a	6.68 ± 0.11a	15.25 ± 0.58b	30.08 ± 1.70b	11.81 ± 0.38b	11.46 ± 0.54b
100 ppm	2.04 ± 0.07ab	1.72 ± 0.08ab	1.33 ± 0.06a	1.83 ± 0.12a	6.93 ± 0.164a	16.43 ± 0.80b	38.27 ± 3.14a	10.35 ± 0.38c	8.66 ± 0.52c

Within the same column, different letters indicate significant differences at *P* < 0.05 level (one-way ANOVA followed by Tukey HSD tests).

### Transgenerational effects of cucurbitacin B on F_1_ generation of *Aphis gossypii*

The young instars development duration and adult longevity of the F_1_ generation results are shown in Table 1. When parental generation (F_0_) was exposed to 25 ppm of cucurbitacin B, the duration of 1^st^ instar of F_1_ decreased (*F* = 6.36*;* df = 2,178*; P* = 0.002) and the duration of 2^nd^ instar increased significantly (*F* = 4.37; df = 2,178; *P* = 0.014). Similarly, when the F_0_ was exposed to 100 ppm of cucurbitacin B, the duration of 3^rd^ instar of F_1_ also increased significantly (*F* = 7.17; df = 2,178; *P* = 0.001). No statistical differences were observed on the duration of 4^th^ instar (*F* = 0.33; df = 2,178; *P* = 0.714) and the total immature development duration (*F* = 1.65; df = 2, 178; *P* = 0.194) after treated by both concentrations of cucurbitacin B in comparison with control. All these effects of cucurbitacin B on developmental stages of melon aphid of F_1_ generation showed the treatment of cucurbitacin B on F_0_ only exhibited some influences on early life stages of F_1_ melon aphid.

**Figure 2:**
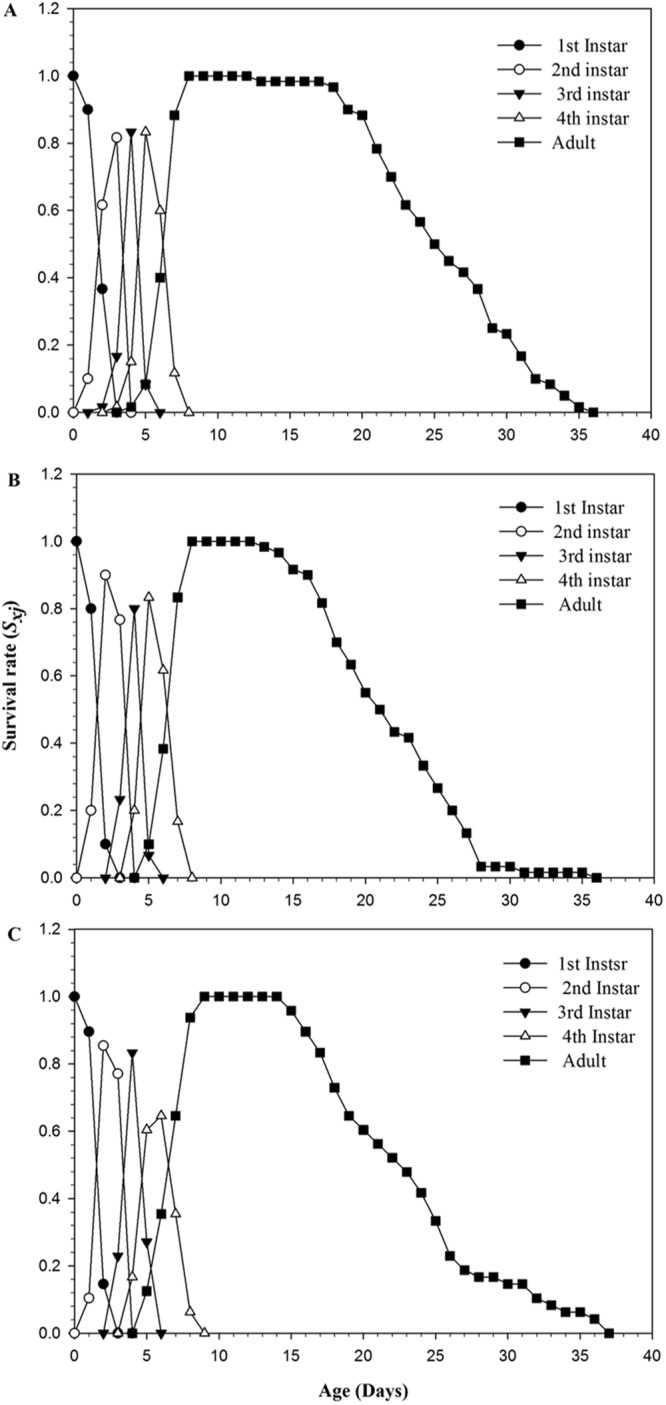
Age-stage specific survival rate (*s*_xj_) of *A*. *gossypii* individuals belonging to the F_1_ generation descending from parents (F_0_) under untreated control conditions (**A**), treated with 25 ppm (**B**) and 100 ppm (**C**) of cucurbitacin B.

Transgenerational effects of 25 and 100 ppm of cucurbitacin B on population dynamics were estimated with a paired bootstrap test using TWOSEX MS chart program^49^ based on life table of F_1_ generation. It was found that the population dynamics parameters of F_1_ generation, such as the intrinsic rate of increase *r* (day^−1^), the finite rate of increase *λ* (day^−1^) and the mean generation time *T* (day) though decreased at 25 ppm concentration as compared to control population, however, the net reproductive rate of increase *R*_0_ (offspring/individual) decreased significantly at 25 ppm concentration. While at 100 ppm concentration, the net reproductive rate of increase *R*_0_ (offspring/individual) and the mean generation time *T* (day) decreased, however not significantly as compared to control (Table 2). The age-stage specific survival rate (*s*_xj_) (Fig. 2) showed the probability that newborn nymphs will survive to age x and stage j. The newborn nymphs from the control (Fig. 2A), 25 ppm (Fig. 2B) and 100 ppm (Fig. 2C) treatments exhibited variable developmental rates in juvenile stages, moreover, the different immature stages overlapped with each other. However, the adult survival rate is different for the control, 25 ppm and 100 ppm group. The declined survival rate of adults was recorded at the 13^th^ and the 14^th^ day of adult stage respectively in 25 ppm and 100 ppm group, while the decline of survival rate occurred at the 18^th^ day of adult stage in control (Fig. 2A). These indicated that the cucurbitacin B exposed F_1_ melon aphid groups were less stable as compared to control group.

**Table 2:** Estimated population growth parameters for aphids belonging to the F_1_ generation descending from parents (F_0_) exposed to 25 and 100 ppm of cucurbitacin B, compared to the untreated control population.

Parameters	Bootstrap					
	Control	25 ppm	*P*	Control	100 ppm	*P*
*r* (day^−1^)	0.2677 ± 0.006a	0.2582 ± 5.133a	0.257	0.2677 ± 0.006a	0.2657 ± 6.554a	0.843
*λ* (day^−1^)	1.3070 ± 8.559a	1.2946 ± 6.646a	0.257	1.3070 ± 8.559a	1.3044 ± 8.549a	0.843
*R*_0_ (offspring/individual)	40.0639 ± 2.393a	30.0833 ± 1.684b	<0.001	40.0639 ± 2.393a	38.2708 ± 3.111a	0.652
*T* (day)	13.7881 ± 0.287a	13.1895 ± 0.276a	0.12	13.7881 ± 0.287a	13.7068 ± 0.397a	0.871

*r*: intrinsic rate of increase (day^−1^), *λ*: finite rate of increase (day^−1^), *R*_o_: net reproductive rate (offspring/individual), *T*: mean generation time (day) were estimated by using 100,000 bootstraps replications.

Within the same row, different letters indicate significant differences between the control and the cucurbitacin B concentration groups (significant at the *P* < 0.05 level, paired bootstrap test using TWOSEX MS chart program).

**Figure 3:**
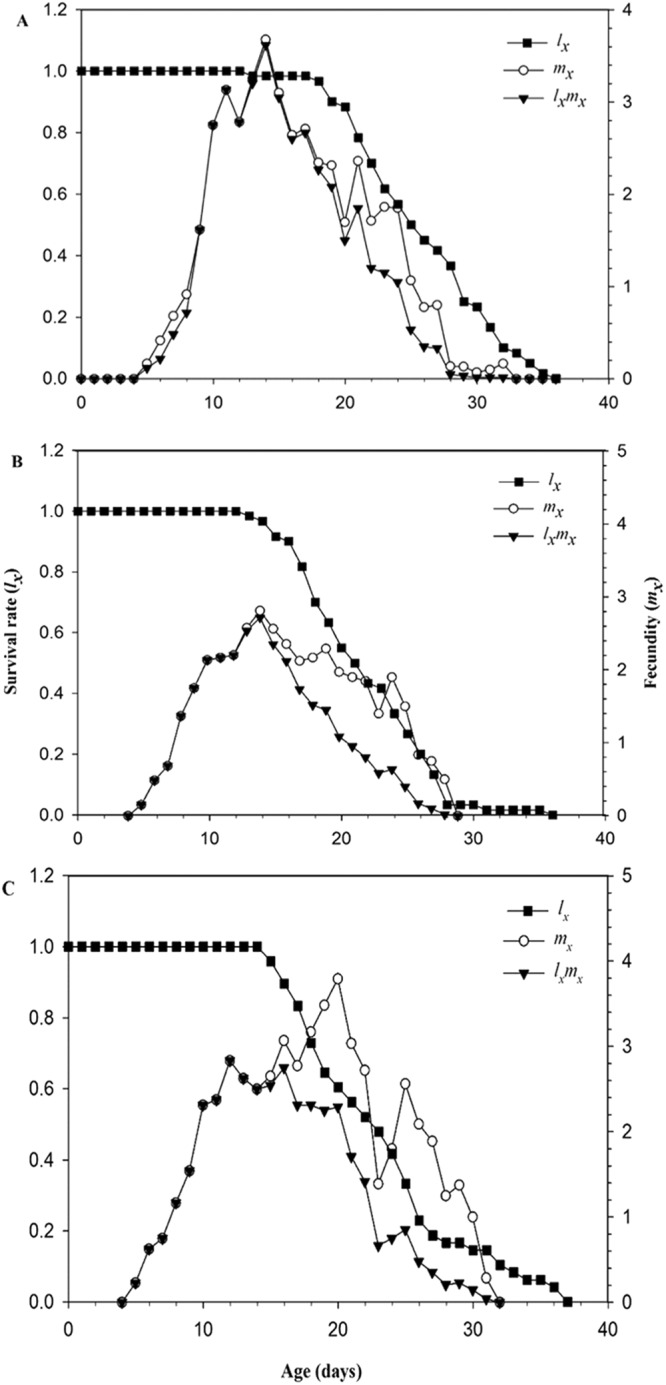
Population age-specific survival rate (*l*_x_), age-specific fecundity (*m*_*x*_) and the age-specific maturity (*l*_*x*_*m*_*x*_) of *A*. *gossypii* of the F_1_ generation descending from parents (F_0_) exposed to 25 (**B**) and 100 ppm (**C**) of cucurbitacin B, compared to the untreated control (**A**) population.

The population age-specific survival rate (*l*_x_) declined rapidly in both treatments of 25 and 100 ppm of cucurbitacin B (Fig. 3B,C) as compared to control. In control, the population started to decline after 18 days (Fig. 3A), whereas in treatments of 25 and 100 ppm of cucurbitacin B, the population started to decline on the 13^th^ and 14^th^ day respectively (Fig. 3). This indicated that the survival probability of newborn nymphs of the two treatments groups was less as compared to control group. The curves of age-specific fecundity (*m*_*x*_) showed that the reproduction of melon aphid began at same time in all concentrations (Fig. 3). However, the ovipositional period lasted at 33 days in control (Fig. 3A), while in 25 and 100 ppm the ovipositional period lasted at 29 and 32 days respectively (Fig. 3B,C). Similarly, the age specific maturity (*l*_*x*_*m*_*x*_) started to decline at 15^th^ day in control (Fig. 3A) population, whereas in 25 and 100 ppm of cucurbitacin B, the age specific maturity (*l*_*x*_*m*_*x*_) started to decline at 14^th^ and 12^th^ day respectively (Fig. 3B,C).

**Figure 4:**
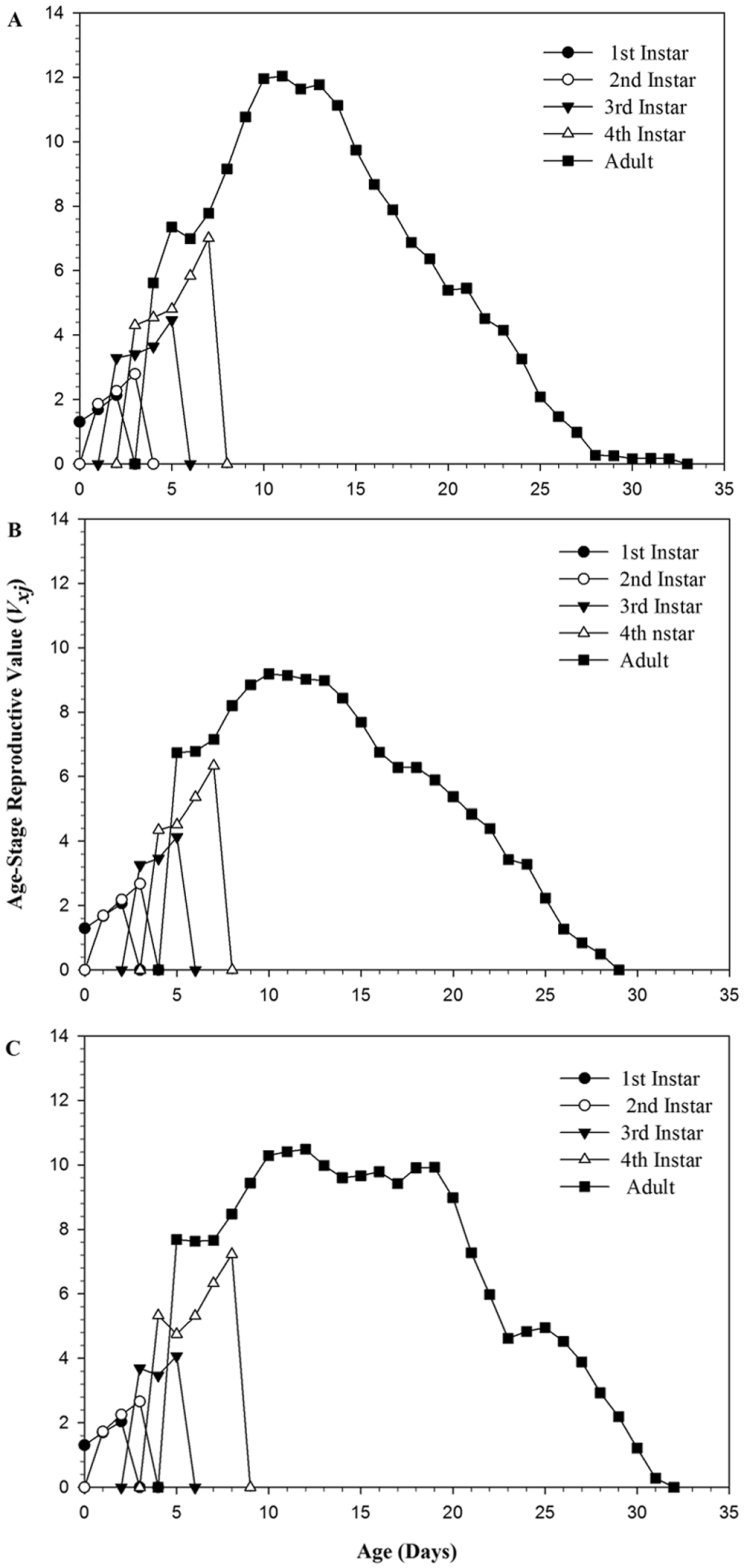
Age-stage reproductive value (*v*_xj_) of *A*. *gossypii* individuals belonging to the F_1_ generation descending from parents (F_0_) under control conditions (**A**), treated with 25 ppm (**B**) and 100 ppm (**C**) of cucurbitacin B.

The age-stage reproductive value (*v*_xj_) of melon aphid showed that the *v*_xj_ of 25 (Fig. 4B) and 100 ppm (Fig. 4C) of cucurbitacin B treated adults was different with the adults of the control group (Fig. 4A). On the one side, the *v*_xj_ of 25 and 100 ppm cucurbitacin B treated adults decreased as compare to control, for example, the maximum *v*_xj_ value for control was 12.0 at age of the 11^th^ day, which was higher than the maximum *v*_xj_ value 9.5 at age of 10^th^ day in 25 ppm treated adults and 10.2 at age of 12^th^ day in 100 ppm treated adult. (Fig. 4). On the other side, the reproductive duration of F_1_ adults also changed after F_0_ exposed to 25 and 100 ppm of cucurbitacin B. It was found that in control, the reproductive duration of F_1_ adults was 9 days with the *v*_xj_ value is more than 8, while the reproductive duration of 25 and 100 ppm was 7 and 13 days with the *v*_xj_ value is more than 8, respectively. This indicated that 25 ppm suppressed the reproductive duration of F_1_ adult while 100 ppm of cucurbitacin B increased the reproductive duration of F_1_ adults, in comparison to control. The age-specific survival rate (*l*_x)_ and the age-stage reproductive (*v*_xj_) curves showed that both 25 and 100 ppm cucurbitacin B mainly affected the adult stages of F_1_ melon aphid (Figs 3 and 4).

## Discussion

In this study we investigated the direct acute toxicity of six increasing concentrations the allelochemical, cucurbitacin B, on adults of the melon aphid. The results showed that the mortality of adults of the melon aphids increased with increasing cucurbitacin B concentrations. A similar trend has also been reported for spider mite (*T*. *urticae*) exposed to cucurbitacin C^22^. The present results showed that cucurbitacin B at 800 ppm resulted in 67.47% mortality of melon aphid, while Kamel and El-Gengaihi^47^ found only the 40% mortality of *A*. *gossypii* after exposure to leaves that have been dipped into 2000 ppm of cucurbitacin B. The difference between the two results might be mainly due to the different exposure methods used. We included cucurbitacin B into the artificial diet, thus we ensured that the chemical was present in the feeding substrate (i.e., efficient exposure), while in the previous study by Kamel and El-Gengaihi^47^, there is no clear evidence that cucurbitacin B entered into the leaves tissues.

More interesting results were obtained when characterizing the sublethal effects of cucurbitacin B at the demographical level of *A*. *gossypii*. Feeding exposure to cucurbitacin B at 25 ppm, and even more markedly at 100 ppm, significantly reduced the adult longevity and fecundity of F_0_ generation as compared to the control group. This result agrees with that of the longevity and fecundity of the cabbage aphid significantly reduced when exposed to an increased concentration of cucurbitacin B^50^. Azadirachtin, (triterpene) significantly reduced the longevity and fecundity of *A*. *gossypii* with an increase in concentration^51^. Additionally, the fitness of *B*. *tabaci* reduced with increasing concentrations of the phenolic aldehyde^52^, gossypol, from plants of the genus *Gossypium*. High gossypol concentration reduced the fecundity and growth rate of beet armyworm, *S*. *exigua*^53,54^. All these results indicated that the dose-dependent detrimental effects of plant secondary metabolites including cucurbitacin B on insect’s biological traits can be widely diffused in various host plants-herbivore complexes.

When investigating at the transgenerational level, i.e., the effects on the progeny (F_1_) of the treated adults (F_0_), we noticed a decrease in longevity and fecundity. However, there was no evident dose-response effect, because the F_1_ of adults exposed to 25 ppm of cucurbitacin B showed a decrease in both longevity and fecundity, while the progeny of adults exposed to 100 ppm of the allelochemical had only a decreased longevity. The reduction of F_1_ fecundity in the treatment of 25 ppm of cucurbitacin B was due to the short longevity and low age-stage reproductive value (*v*_xj_), while the no change of F_1_ fecundity in 100 ppm of cucurbitacin B treatment was due to the combined effects of the increased reproductive duration of adults with the decreased longevity and the reproductive value (*v*_xj_). To better understand the potential dose dependent effects at the transgenerational level, more concentrations should be tested. However, the two concentrations of cucurbitacin B exhibited different effects on F_1_ demographical parameters. The results showed that after F_0_ was exposed to 25 ppm cucurbitacin B, all the population characteristics of F_1_ generation, including intrinsic rate of increase *r* (day^−1^), finite rate of increase *λ* (day^−1^) and generation time *T* (day) decreased as compared to control, however, only the net reproductive rate *R*_0_ (offspring/individual) decreased significantly at 25 ppm of cucurbitacin B. Moreover, for the treatment of 100 ppm, the valuse for net reproductive rate of increase *R*_0_ (offspring/individual) and the mean generation time *T* (day) of F_1_ were lower than the control.

Negative effects at the demographical level of the exposed individuals have been reported following exposure to other plant secondary metabolites, such as the neem-based insecticide, azadirachtin, on *A*. *gossypii*, *Acyrthosiphon pisum* and *Myzus persicae*^51,55,56^, and after exposure to gossypol in *S*. *exigua* and *B. tabaci*^52,53^. Therefore, the detrimental effects of plant derived chemicals on insect population extensively existed and this is undoubtedly related to the defensive roles against herbivory of plant secondary metabolites^57^.

In contrast to all these results, there are experimental evidences that cucurbitacins could act as phagostimulants for certain herbivore insects, such as chrysomelid beetles, e.g., *Ceratoma* spp., *Acalyma* spp. and *Diabroticina* spp. These beetles were found preferring cucurbitacin containing plants because cucurbitacin could protect them from their predator and parasites^58–60^. Moreover, the eggplant lace bugs, (*Gargaphia solani*) and the sycamore lace bugs (*Corythucha ciliata*) preferred diets that contain cucurbitacin B^20^. Based on the phagostimulant property of cucurbitacins to some insects, the mixture of cucurbitacin with insecticides has been developed as bait to insects and was used in IPM program of these pests^61,62^.

Overall, we can conclude that this study supports the hypothesis that host plants containing high levels of cucurbitacin B have the potential to defense themselves from the feeding activity of an important sap-sucking insect pest, the melon aphid, by impairing its biological fitness sublethally. Therefore, plant breeding should take into account this important trait as one of the criteria used for the selection of new varieties to be incorporated into IPM programs. However, further studies are needed to better understand (i) how and when the cucurbitacin content in the plants can be enhanced, and (ii) how plants with high cucurbitacin content can be integrated with other IPM tools, such as aphid natural enemies, that can be affected by this allelochemical via their prey/hosts^63,64^, and with insecticides. For instance, it is well known that insect metabolic enzymes (e.g., P450s and glutathione-S-transferase), that are more active in pest populations resistant to certain insecticides, can play a key role in the detoxifying plant secondary metabolites^65^.

## Materials and Methods

### Insects and Cucurbitacin B

The stock culture of melon aphid was collected from cucumber plots planted at the China Agricultural University, Beijing, China, during spring, 2016. The stock culture was reared in the laboratory (25 ± 1 °C; 75% RH; 16:8 L: D) for one year on cucumber plants without exposure to any pesticide. Cucurbitacin B (92% of purity) was obtained by purification the crude cucurbitacin B (60% of purity, from Tianjin Chemical Co.) with silica column chromatography (R_f_ = 0.65, 10:2 of ethyl acetate-petroleum ether(b.p.70 °C)), and was characterized by ^1^H NMR (300 MHz, CDCl_3_):0.98(H-18), 1.08(H-19), 1.28(H-29), 1.35(H-28), 1.38(H-30), 1.44(H-21), 1.54(H-26), 1.57(H-27), 1.96(H-7), 1.98(H-8), 2.01(O_2_CCH_3_), 2.48,2.50(d,H-17), 2.66,2.71,3.22,3.26(H-12), 4.25(H-16), 4.41(H-2), 5.78,5.79(H-6), 6.44,6.49(H-23), 7.04,7.09(H-24). The purity of the finale cucurbitacin B was determined by HPLC with 98% cucurbitacin B (Sigma-Aldrich) as standard for comparison.

### *Aphis gossypii* exposure to cucurbitacin B and concentration-mortality response bioassay

Around 450 apterous adult aphids were released on healthy cucumber plants. After 24 hours, all adult aphids were removed except newborn nymphs. The latter were allowed to grow and become adults. In about 8 days, most of the newborn nymphs passed all growth stages and become adults^30^. This procedure was used to ensure that all the aphids to be used in the experiments were of the same life instar and coetaneous.

For allowing aphid feeding on various concentrations of cucurbitacin B, we contaminated an artificial diet with slight modifications^66,67^. The mixture of artificial diet, containing 17% sucrose and cucurbitacin B (at six concentrations: 25, 50, 100, 200, 400 and 800 ppm), was sealed between two layers of parafilm and covered in one side by a 4-cm diameter feeding arena. The other side of the arena was covered with a fine mesh of Chinese art paper to prevent their escape. Aphids used for the bioassays were starved for four hours prior to release in the test arenas. Thirty young adult aphids, obtained as described above, were released into each arena and all the experiments were replicated three times. Mortality of melon aphids was assessed 48 h after feeding the cucurbitacin B, aphids were considered dead when they did not react after being touched with a fine paint brush.

### Effects of cucurbitacin B on life history traits of melon aphid F_0_ and F_1_ generations

The basal level of cucurbitacin B in plants varies among plant species, and even within the same plant species under different growth conditions. While 50 mg/kg of cucurbitacin B have been found in *Cucumis asper* leaves^68^, the cucurbitacin B contents has been detected as 23.8 μg/mL, 9.6 μg/mL, 4.3 μg/mL, 2.7 μg/mL, 2.5 μg/mL and 1.3 μg/mL in *Momordica charantia*, *C*. *sativus*, *C*. *melo* var. conomon, *F*. *albus*, *Vigna sesquipedalis*, *Benincasa hispida* var. chieh-qua and *Luffa cylindrical*, respectively^48^. Although specific studies are still lacking, the synthesis of cucurbitacin B could be induced by herbivory, so its concentration could increase after aphid feeding, as demonstrated for other allelochemicals (phenols) in okra plants where *A*. *gossypii* had feed^69^. In this context, we chose a high and a very high concentration of cucurbitacin B, i.e, 25 and 100 ppm (which caused 25.1% and 39.25% mortality respectively, see the result section) for testing the effects on the demography of adults of the exposed generation (F_0_) and in juveniles and adults of their progeny (F_1_). For this, we followed the same experimental setup described above, with the exceptions that fifty healthy adult aphids were placed into each arena and that the experiment was repeated four times. The control group was fed with only artificial diet without cucurbitacin B. After 48 hours, sixty survived and healthy aphids were collected for each cucurbitacin B treatment and for the control group. Aphids, pretreated with 25, 100 ppm of cucurbitacin B and control, were individually placed on 20 mm diameter insecticide-free leaf discs. The leaf discs were placed on each agar bed (1.5 mL of 2% (w/v) agar) in wells of 12-well cell culture plates and covered with filter paper to prevent aphid escape. New cucumber leaf discs were replaced every 3^rd^ day during the experiment. In order to eliminate the effects of cucurbitacin B in cucumber leaf disc on the biological parameters of melon aphids, the leaf in the same position of cucumber seedlings with similar size was used for preparing the leaf discs including the control leaf discs. Moreover, it was found cucurbitacin B present only in very young seedlings and nearly not in older leaves in cucumber (*C*. *sativus*)^68^, then the effects of leaf replacement on the biological parameters of melon aphid could be ignored. All cohorts from the treatment groups of 25, 100 ppm and the control were reared under the laboratory conditions (25 ± 1 °C, 75% RH, 16:8/L:D) and observed daily. To assess the effect of cucurbitacin B on F_0_ generation of melon aphid, newborn nymphs were removed and the adult longevity and fecundity was recorded until adults died.

To assess the effect of cucurbitacin B on the F_1_ generation, the same method and treatments were used as in the case of F_0_ generation. All aphids from the control and the treatment groups were placed on insecticide free cucumber leaf disc individually and then transferred to 12-well cell-culture plates containing 2% (w/v) agar and covered with filter paper. When aphid started reproducing, adult aphids were removed and only one nymph was left in each leaf disc and was used as F_1_ generation. This procedure was repeated 60 times for control and treatment groups using single aphid as replication. Nymphs were transferred to insecticide free leaf discs every 3^rd^ day, and when aphid started reproducing, all nymphs were counted and removed on a daily basis until adult died. Biological parameters including development, longevity and fecundity of F_1_ generation were recorded daily.

### Data Analyses

Corrected mortality of melon aphids was determined by using the Abbot’s formula^70^. The statistical differences among data related to adult longevity, fecundity, duration of instars and duration of immature stage of melon aphids were analyzed by one-way ANOVA followed by the Tukey post hoc test (*P* < 0.05) (IBM, SPSS Statistics). Life table data of melon aphid were analyzed according to an age-stage, twosex life table^45,46^ using the TWOSEX-MS Chart computer program^49^. The population age-specific survival rate (*l*_x_), age-specific fecundity (*m*_*x*_), the age specific maturity (*l*_*x*_*m*_*x*_), the age-stage specific survival rate (*s*_xj_), the age-stage reproductive value (*v*_xj_)^71,72^, the net reproductive rate *R*_0_ (offspring/individual), intrinsic rate of increase *r* (day^−1^), the finite rate of increase *λ* (day^−1^) and mean generation time *T* (day) were calculated according to Chi and Liu^45^. Using the TWOSEX-MS Chart computer program, the bootstrap technique^73^ was used to calculate the means and standard error. For bootstrap, 100,000 replicates were used^74,75^. Fecundity, survival rate and reproductive value curves were generated using Sigma Plot 12.0 (Systat Software Inc., San Jose, CA).

## Data Availability

All data analyzed during this study are available from the corresponding author on reasonable request.
